# The critical importance of the fetal hypothalamus-pituitary-adrenal axis

**DOI:** 10.12688/f1000research.7224.1

**Published:** 2016-01-28

**Authors:** Charles E. Wood, Maureen Keller-Wood

**Affiliations:** 1 Department of Physiology and Functional Genomics, University of Florida College of Medicine, FL, USA; 2 Department of Pharmacodynamics, University of Florida College of Pharmacy, FL, USA

**Keywords:** fetal hypothalamus-pituitary-adrenal axis, HPA, cortisol

## Abstract

The fetal hypothalamus-pituitary-adrenal (HPA) axis is at the center of mechanisms controlling fetal readiness for birth, survival after birth and, in several species, determination of the timing of birth. Stereotypical increases in fetal HPA axis activity at the end of gestation are critical for preparing the fetus for successful transition to postnatal life. The fundamental importance in fetal development of the endogenous activation of this endocrine axis at the end of gestation has led to the use of glucocorticoids for reducing neonatal morbidity in premature infants. However, the choice of dose and repetition of treatments has been controversial, raising the possibility that excess glucocorticoid might program an increased incidence of adult disease (e.g., coronary artery disease and diabetes). We make the argument that because of the critical importance of the fetal HPA axis and its interaction with the maternal HPA axis, dysregulation of cortisol plasma concentrations or inappropriate manipulation pharmacologically can have negative consequences at the beginning of extrauterine life and for decades thereafter.

Cortisol is arguably the most important hormone for organizing maturation in late gestation. The discovery that, in sheep, interruption of the fetal hypothalamus-pituitary-adrenal (HPA) axis prevents spontaneous parturition ^1, 2^ initiated a series of important studies on the role that cortisol plays in the coordination of fetal readiness for extrauterine life. The mechanism of parturition and the role that the fetal adrenal plays in primate species are complicated compared with ruminants and other large animals. But, although cortisol does not directly initiate parturition in the human, it does have other critically important actions in the human: actions that were first discovered in the sheep model. Perhaps the most impactful discovery was that fetal sheep that were premature but otherwise treated with glucocorticoid were less likely to die in respiratory distress ^3^. The mechanism of this effect involves a direct action of glucocorticoid on the fetal lung, accelerating terminal development and stimulating the production of pulmonary surfactant ^4– 6^. This discovery, rapidly translated to the human ^7, 8^, has resulted in the widespread antenatal administration of glucocorticoids to pregnant women threatening premature labor ^9, 10^. This clinical practice has been an effective strategy for reduction or elimination of ventilator assist of the premature infant after birth. In addition to its effect on the lung, cortisol plays an important role in accelerating development of the fetal gastrointestinal tract ^11^ and liver ^12^. Recent evidence also supports a role for cortisol in the development of the fetal cardiovascular system ^13^ and heart ^14^. Similar to its effect in other systems, cortisol helps ready the fetal cardiovascular system for the transition to extrauterine life.

A central tenet of endocrinology is that circulating concentrations of hormones are controlled: that there is a set point around which negative feedback mechanisms maintain concentrations within a range ^15, 16^. For a hormonal system as important as the fetal HPA axis, understanding those mechanisms that govern the increases in fetal adrenocorticotropic hormone (ACTH) and cortisol becomes critically important for understanding the timing of birth, readiness for birth, and survival of the newborn. This most often involves the elucidation of influences, both positive and negative, that determine the trajectory of fetal ACTH secretion. For example, Thorburn and colleagues discovered a dynamic increase in the sensitivity of the fetal adrenal cortex to ACTH that can account for much of the increase in circulating cortisol concentrations prior to birth ^17^. Several laboratories have investigated changes in circulating molecular forms of immunoreactive ACTH (resulting from partial or complete processing of the parent molecule pro-opiomelanocortin), altering the effective biological activity of this pituitary hormone ^18– 21^. New evidence suggests that peripheral interconversion of cortisol and cortisone is also an important variable that can contribute to changes in circulating concentrations of cortisol ^22^. Balancing the stimulation of the axis are mechanisms that prevent over-activity of the axis. The late-gestation fetal sheep has a cortisol negative feedback mechanism that is remarkably sensitive to small changes in plasma cortisol concentration ^23– 27^. In the final days and hours of fetal life, this negative feedback sensitivity falls dramatically, allowing greater increases in fetal HPA axis activity ^28^.

Although nearly every variable within the fetal HPA axis has been studied to some extent, we are not close to fully understanding the interplay between the normal fetal developmental patterns, the influence of fetal and maternal stress, and the modulatory influence of infection on the timing of parturition and the survival of the newborn. A danger in the translation of basic endocrinology to clinical practice has been an under-appreciation of untoward actions of hormones when they are present in unnaturally high concentrations. A clear example of this is the use, in practice, of antenatal steroid administration to women threatening preterm labor. First approved by a National Institutes of Health (NIH) consensus panel as a single treatment or a small number of repeated treatments ^7^, the procedure was modified by practicing physicians so as to administer glucocorticoid weekly from as early as 22 to 24 weeks’ gestation ^29^. Forgetting the basic rule of endocrinology—that control mechanisms prevent excessively high, as well as excessively low concentrations of hormones—can create unintended problems. Weekly or biweekly antenatal maternal betamethasone treatment resulted in the birth of babies that were more likely to be growth-restricted ^29– 31^. Studies in animal models indicate that multiple doses of glucocorticoid can have negative neurodevelopmental outcomes. Using the sheep model, Newnham and colleagues clearly demonstrated that excessive treatment with glucocorticoid dramatically impacts fetal brain growth and development ^32, 33^. This laboratory and others have also demonstrated negative effects of excessive glucocorticoid on fetal somatic growth ^34, 35^. Recent evidence in the baboon model reveals sex-specific effects of antenatal betamethasone on learning and attention in the offspring ^36^. Recent work has suggested that the effects of excess glucocorticoid can be codified in the epigenome of the infant, theoretically with effects that could last a lifetime ^37, 38^. On balance, however, the long-term biological cost of multiple treatments is not clear. Clinical trials have not demonstrated long-term growth or major neurosensory disabilities but have indicated an increased likelihood of attention deficit in children who were exposed to multiple doses of glucocorticoid before birth ^39^. Arterial stiffness, however, was increased in 14- to 26-year-old subjects exposed to two to nine weekly doses of betamethasone ^40^. In recognition of the potential risks of multiple antenatal glucocorticoid treatments, a second NIH consensus statement recommended a single treatment ^41^. As a result of the revised recommendations, the incidence of multiple treatment has been reduced.

The fetus is an organism that is distinct from, yet dependent on, its mother. The circulation of the fetus does not admix with the circulation of the mother, and the fetus in late gestation has the capacity to synthesize its own hormones. However, by virtue of the fact that the fetal and maternal blood is separated by one or more layers of cells in the placenta (depending on the species and therefore the general structure of the placenta), there is a molecular communication between mother and fetus. This communication is most obvious with regard to blood gases. Fetal growth and development depend on the oxygen and nutrients supplied by the mother via trans-placental passage. However, in addition to hormones synthesized within the placenta (e.g., chorionic gonadotropin and chorionic somatomammotropin), there is endocrine communication. An important example of this communication is the influence of maternal adrenal cortical hormones on the fetus, perhaps a mechanism by which the fetus is informed about maternal stress.

The placentas of many species, including the human, rodent species, and the sheep, express the enzyme 11β-hydroxysteroid dehydrogenase types 1 and 2 (encoded by the genes *HSD11B1* and *HSD11B2*), which interconvert cortisol and cortisone (or, in rodent species, corticosterone and 11-dehydrocorticosterone) ^42– 45^. The predominant reaction in placenta is oxidation (cortisol to cortisone and corticosterone to 11-dehydrocorticosterone, mediated by the type 2 enzyme isoform). Because cortisone and 11-dehydrocorticosterone have low affinity for the glucocorticoid and mineralocorticoid receptors (GR and MR, respectively), some of the active glucocorticoid in the maternal circulation is inactivated upon passage into the fetal circulation. Although this is a partial barrier for the natural glucocorticoids, it is not complete. Some of the cortisol that was converted to cortisone upon transplacental passage is converted back to cortisol by the type 1 11β-HSD isoform in target tissues, such as lung ^46, 47^, brain ^48^, and heart ^49– 51^. As shown in the chronically catheterized fetal sheep model, elevation of maternal cortisol concentration to levels typical of stress increases fetal plasma cortisol concentrations and inhibits fetal ACTH secretion via a negative feedback mechanism ^52^. Synthetic glucocorticoids, such as dexamethasone or betamethasone, are not substrates for 11β-HSD and therefore are not inactivated when passing through the placenta.

The passage of natural glucocorticoid across placenta from mother to fetus raises the question of whether maternal stress is detrimental to the fetus. There is recent interest in the potential “programming” effects of maternal stress on the pattern of development in the fetus ^53^. There are known glucocorticoid effects on gene methylation and histone modification that can have long-lasting effects on the physiology of the offspring. Perinatal programming of the HPA axis has been reviewed elsewhere ^54^. If “programming” can result from alterations in the genome, however, can it also result from a more immediate effect of hypercortisolemia that bends the arc of development? In other words, what are the more immediate consequences of maternal stress?

The literature is rife with reports of poor pregnancy outcome when the pregnancy is complicated by maternal stress: low socioeconomic class, partner abuse, and violence ^55– 57^. It is not known whether these negative effects on the pregnancy are caused entirely, or even partly, by the maternal cortisol stress response, but there is evidence that suggests that cortisol is at least partly to blame. For example, women with Cushing disease have a higher incidence of stillbirth that is associated with obstructive hypertrophic cardiomyopathy ^58^. The effect of the maternal hypercortisolemia can be reproduced in the sheep model (using cortisol administration rates that mimic the maternal stress response) ^59^. However, maternal cortisol also has an effect on fetal somatic growth, in part secondary to alterations in uterine blood flow ^60^. Increases in maternal cortisol also alter fetal pulmonary and renal fluid balance mechanisms ^61^. How many of these modifications of the pattern of fetal growth and development result in increased incidence of morbidity and mortality in postnatal life? For example, does chronic maternal stress cause small-for-gestation babies ^62^ that are more prone to infant mortality ^63^ or metabolic disease in adulthood? Does cortisol-induced alteration in fetal cardiac development underlie the increased incidence of coronary artery disease in adults who were small for gestational age at birth ^64^?

As it is detrimental to chronically elevate maternal plasma cortisol concentration, it is important to remember that there is a physiological set point for cortisol in maternal blood during pregnancy, and that reductions in maternal cortisol below that level could be detrimental. Circulating concentrations of cortisol in the human and in other species, such as the sheep, are naturally increased in the latter half of pregnancy ^65– 67^. Stress, as previously discussed, further elevates the concentration of cortisol ^68, 69^. But the fact that the normal set point for cortisol is increased in pregnancy raises the question of why this occurs and whether there are species differences with regard to mechanism ^68^.

The normal pregnancy-associated increase in maternal adrenal secretion of cortisol appears to play an important role on both sides of the placenta. Prior to the final stages of fetal life *in utero*, the majority of the cortisol circulating in the fetal blood derives from the maternal adrenal glands ^70^. Because cortisol plays an important role in developmental processes important for fetal readiness for birth, it is likely that the increase in maternal cortisol plasma concentrations provides an important source of cortisol to the fetus before the fetal HPA axis becomes fully competent. On the other hand, reduction in maternal cortisol concentration below the normal set point, down to the level normally observed in the nonpregnant state, disturbs blood pressure, fluid balance, and uterine blood flow, and slows fetal growth ^66^. Although the above-mentioned studies were performed in sheep, evidence from human pregnancies is consistent: untreated maternal adrenal insufficiency causes premature labor and neonatal morbidity ^71, 72^.

It is important to recognize both the importance and the complexity of the HPA axis during pregnancy and during fetal development. The mechanisms controlling this endocrine axis during pregnancy (why is maternal cortisol normally increased during late gestation?) and fetal life (what causes the increase in fetal HPA axis activity that is important for neonatal survival?) have never been fully solved. For example, is the ontogenetic rise in fetal HPA axis activity caused secondary to the development of immune cells within the fetal brain ^73^? Are there placental signaling molecules (e.g., Corticotropin Releasing Hormone [CRH **]** in primate species) that stimulate fetal HPA axis activity ^74^? Are there programmed developmental events within the fetal brain that progress without modification by circulating endocrine signals? We argue that answering these basic questions will be important for reducing neonatal mortality and morbidity, for improvement of maternal health, and the design of better and smarter treatments for women threatening preterm labor, and for the babies that are admitted to neonatal intensive care units.
